# Immunogenicity of COVID-19 Tozinameran Vaccination in Patients on Chronic Dialysis

**DOI:** 10.3389/fimmu.2021.690698

**Published:** 2021-06-30

**Authors:** Eva Schrezenmeier, Leon Bergfeld, David Hillus, Joerg-Detlev Lippert, Ulrike Weber, Pinkus Tober-Lau, Irmgard Landgraf, Tatjana Schwarz, Kai Kappert, Ana-Luisa Stefanski, Arne Sattler, Katja Kotsch, Thomas Dörner, Leif Erik Sander, Klemens Budde, Fabian Halleck, Florian Kurth, Victor Max Corman, Mira Choi

**Affiliations:** ^1^ Department of Nephrology and Intensive Care, Charité-Universitätsmedizin Berlin, Corporate Member of Freie Universität Berlin and Humboldt-Universität zu Berlin, Berlin, Germany; ^2^ Berlin Institute of Health at Charité – Universitätsmedizin Berlin, BIH Academy, Clinician Scientist Program Universitätsmedizin Berlin, Berlin, Germany; ^3^ Institute of Virology, Charité -Universitätsmedizin Berlin, Corporate Member of Freie Universität Berlin and Humboldt-Universität zu Berlin, Berlin, Germany; ^4^ German Centre for Infection Research (DZIF), Partner Site Charité, Berlin, Germany; ^5^ Department of Infectious Diseases and Respiratory Medicine, Charité-Universitätsmedizin Berlin, Corporate Member of Freie Universität Berlin and Humboldt-Universität zu Berlin, Berlin, Germany; ^6^ Nierenzentrum Koethen, Koethen, Germany; ^7^ Hausarztpraxis am Agaplesion Bethanien Sophienhaus, Berlin, Germany; ^8^ Institute of Laboratory Medicine, Clinical Chemistry and Pathobiochemistry, Charité-Universitätsmedizin Berlin, Corporate Member of Freie Universität Berlin and Humboldt-Universität zu Berlin, Berlin, Germany; ^9^ Labor Berlin-Charité Vivantes GmbH, Berlin, Germany; ^10^ Department for General, Visceral and Vascular Surgery, Charité-Universitätsmedizin Berlin, Corporate Member of Freie Universität Berlin and Humboldt-Universität zu Berlin, Berlin, Germany; ^11^ Department of Rheumatology and Clinical Immunology, Charité-Universitätsmedizin Berlin, Corporate Member of Freie Universität Berlin and Humboldt-Universität zu Berlin, Berlin, Germany; ^12^ Department of Tropical Medicine, Bernhard-Nocht Institute for Tropical Medicine, Hamburg, Germany

**Keywords:** vaccination, COVID – 19, dialysis, antibody response, SARS – CoV – 2

## Abstract

Patients with kidney failure have notoriously weak responses to common vaccines. Thus, immunogenicity of novel SARS-CoV-2 vaccines might be impaired in this group. To determine immunogenicity of SARS-CoV-2 vaccination in patients with chronic dialysis, we analyzed the humoral and T-cell response after two doses of mRNA vaccine Tozinameran (BNT162b2 BioNTech/Pfizer). This observational study included 43 patients on dialysis before vaccination with two doses of Tozinameran 21 days apart. Overall, 36 patients completed the observation period until three weeks after the second dose and 32 patients were further analyzed at week 10. Serum samples were analyzed by SARS-CoV-2 specific IgG and IgA antibodies ~1, ~3–4 and ~10 weeks after the second vaccination. In addition, SARS-CoV-2-specific T-cell responses were assessed at ~3–4 weeks by an interferon-gamma release assay (IGRA). Antibody and T cell outcomes at this timepoint were compared to a group of 44 elderly patients not on dialysis, after immunization with Tozinameran. Median age of patients on chronic dialysis was 74.0 years (IQR 66.0, 82.0). The proportion of males was higher (69.4%) than females. Only 20/36 patients (55.6%, 95%CI: 38.29–71.67) developed SARS-CoV-2-IgG antibodies at the first sampling, whereas 32/36 patients (88.9%, 95%CI: 73.00–96.38) demonstrated IgG detection at the second sampling. In a longitudinal follow-up at ~10 weeks after the second dose, the proportion of dialysis patients reactive for anti-SARS-CoV-2-IgG decreased to 27/32 (84.37%, 95%CI: 66.46–94.10) The proportion of anti-SARS-CoV-2 S1 IgA decreased from 33/36 (91.67%; 95%CI: 76.41–97.82) at weeks 3–4 down to 19/32 (59.38; 95%CI: 40.79–75.78). Compared to a cohort of vaccinees with similar age but not on chronic dialysis seroconversion rates and antibody titers were significantly lower. SARS-CoV-2-specific T-cell responses 3 weeks after second vaccination were detected in 21/31 vaccinated dialysis patients (67.7%, 95%CI: 48.53–82.68) compared to 42/44 (93.3%, 95%CI: 76.49–98.84) in controls of similar age. Patients on dialysis demonstrate a delayed, but robust immune response three to four weeks after the second dose, which indicates effective vaccination of this vulnerable group. However, the lower immunogenicity of Tozinameran in these patients needs further attention to develop potential countermeasures such as an additional booster vaccination.

## Introduction

The coronavirus disease 2019 (COVID-19) pandemic has led to an urgent need for effective strategies, in particular for patients with kidney failure (KF), who have a high mortality (1). The COVID-19 vaccine Tozinameran (BioNTech/Pfizer) showed protection from 12 days after the first dose (2) and was demonstrated to be 95% effective in preventing COVID-19 (3). Robust SARS-CoV-2 antibody responses ≥7 days after the second dose were detected (4). However, patients with KF have an impaired response to vaccination (5) and the earliest reports on COVID-19 vaccination suggest that mRNA vaccines are immunogenic but some patients on dialysis do not seroconvert (6–8). Patients on kidney replacement therapy were excluded from previous trials. We investigated immunogenicity to COVID-19 vaccination with Tozinameran in 43 patients with chronic kidney disease (CKD) stage 5 and provide a longitudinal follow-up at 10 weeks after a second dose.

## Methods

**Figure 1:**
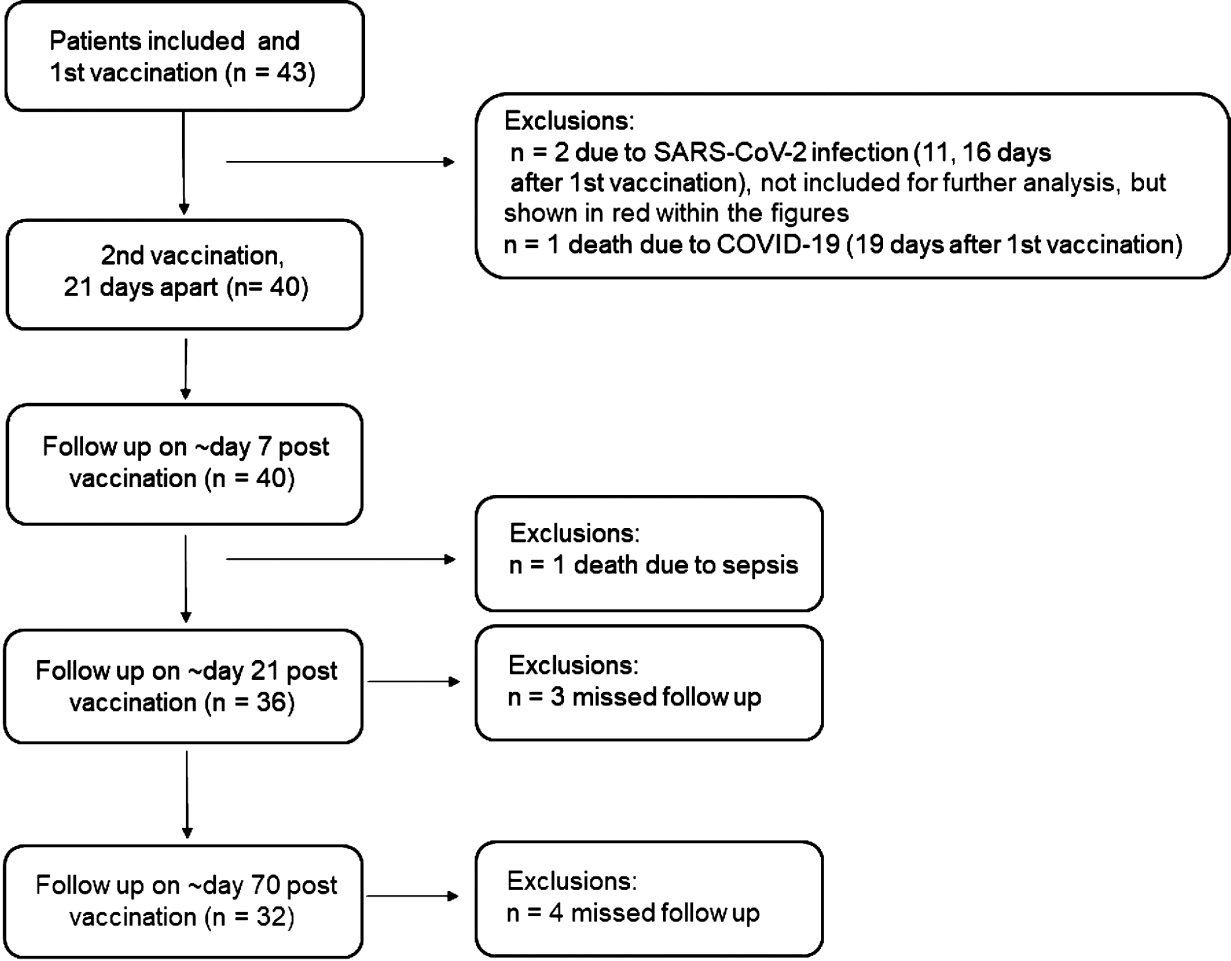
Inclusion diagram dialysis patients.

This study includes 43 patients on dialysis. Samples were taken at three different time points after a second dose of COVID-19 vaccine Tozinameran (BioNTech/Pfizer), which was given 21 days apart: 1st samples were taken on average 7.8 days (range 7–13, week 1), 2nd samples were taken on average 21.2 days (range 20–26, weeks 3–4), and 3rd samples (longitudinal observation) were taken on average 64.7 days (range 64–70, week 10) after the second dose. The study flow diagram is shown in **Figure 1**. Of those two dialysis patients, who were excluded from analysis due to a known prior SARS-CoV-2 infection, we obtained serum samples and marked these in red throughout the figures. In addition to serum samples, full blood (Li-Heparin) for T-cell assays was taken at the 2nd time point. At this time point sero-reactivity was compared to a group of 44 Tozinameran vaccinated persons of similar age but not on dialysis from two ongoing observational cohort studies on immunogenicity of SARS-CoV-2 vaccines (9) and to 18 unvaccinated control patients on hemodialysis.

### Antibody Assessment and Testing of IFN-γ Release of SARS-CoV-2-Specific T Cells

Serum samples of vaccinated dialysis patients were analyzed for anti-SARS-CoV-2 IgG and IgA antibodies ~1, ~3–4, and ~10 weeks after the second dose by anti-SARS-CoV-2-S1 ELISA. ELISA was performed according to the manufacturer’s instructions (Euroimmun Medizinische Labordiagnostika AG, Lübeck, Germany). Briefly, serum samples were analyzed at a 1:101 dilution. Processing and measurement were done using the fully automated Euroimmun Analyzer I (Euroimmun Medizinische Labordiagnostika AG). Optical density (OD) ratios above 1.1 were considered reactive for IgG and IgA (6, 10). At 3–4 weeks after the second doses sero-response was compared to the control cohorts. Two of the unvaccinated control patients on hemodialysis were reported to have RT-PCR confirmed COVID-19 in the past (2 and 3 months before sampling). Comparison was based on a solid phase immunoassay (SeraSpot^®^Anti-SARS-CoV-2 IgG, Seramun Diagnostica, Heidesee, Germany) using four antigens, full spike (fS), spike S1 domain, spike receptor binding domain (RBD), as part of the vaccine, and the nucleocapsid antigen (N), to discriminate between vaccine and SARS-CoV-2 infection induced response. The four antigens and test specific controls are printed in an array arrangement on the bottom of each of the well. Bound antibodies from the patient sera are detected by use of horseradish peroxidase-(HRP)-labeled antibodies against human IgG. Color intensity (SpotSight^®^plate scanner) at the site of formed immune complexes correlates to the antibody concentration. Results were calculated and as normalized signal-to-cutoff (S/CO) ratios by dividing the observed signal strength of a specific spot by that of an internal cutoff control. Samples with an S/CO ratio of ≥1.0 are defined by the manufacturer as reactive.

Potential neutralizing capacity of antibodies was tested by a surrogate virus neutralization test according to the manufacturer´s instructions (cPass Assay, Medac, Wedel, Germany) (11, 12). Briefly, serum samples, positive and negative controls were diluted 1:10 with sample dilution buffer, mixed 1:1 with HRP-RBD solution and incubated at 37°C for 30 min. 100 µl of each sample was added to the hACE2 coated plate and incubation at 37°C for 15 min. After a washing step, TMB solution was added, and the plate was incubated in the dark at room temperature for 15 min. Then, 50 µl stop solution was added per well and the optical density at 450 nm was measured. For calculation of the relative inhibition, the following formula was applied: Inhibition score (%) = (1 − (OD value sample/OD value negative control) × 100%). Samples were considered negative at an inhibition of <30% and positive at an inhibition of ≥30%.

Finally, we applied a commercially available interferon-gamma release assay (IGRA) for assessment of IFN-γ release of SARS-CoV-2-specific T-cell. (Euroimmun Medizinische Labordiagnostika AG, Lübeck, Germany). Here, in parallel 0.5 ml freshly collected lithium-heparin blood (~4 h after blood sampling) was stimulated with a SARS-CoV-2 peptide pool from the spike domain, 0.5 ml of blood was stimulated with mitogen as a positive control (**Supplementary Figure S4**), and 0.5 ml of blood in a blank as a negative control. After 24 h of incubation (at 37°C) IFN-γ concentration in the plasma fraction of all three stimulation tubes was measured by ELISA. IFN-γ response in the blank served as a measure of patient-individual background IFN-γ activity and was subtracted from IFN-γ response in the stimulation tubes. Only samples showing an IFN-γ activity of at least 100 mIU/ml in the mitogen stimulated tube were further analyzed.

### Ethical Statement

This study was approved by the local institutional review board of the ethics committee of Charité-Universitätsmedizin Berlin, Germany (approval number EA4/188/20) and by the local ethics committee of Sachsen-Anhalt (EA7/21). The testing of non-dialysis control subjects is part of ongoing studies on SARS-CoV-2 infection and immunogenicity of COVID-19 vaccines in elderly people and healthcare workers under Charité ethical review board file numbers EA1/068/20, EA4/244/20, and EA4/245/20 (9). Written informed consent was obtained from all patients and healthy volunteers before inclusion in the studies. The studies were carried out in line with the guidelines of Good Clinical Practice (ICH 1996) and the Declaration of Helsinki.

### Statistics

Statistical analyses were performed using GraphPad Prism Version 9. Measurements were tested for normal/lognormal distribution prior to analysis. Differences between groups that deviated significantly from Gaussian distribution were assessed by Mann‐Whitney U test (two groups) or Kruskal–Wallis test (more than two groups) and paired samples (repeat measurements) were compared using the Wilcoxon matched-pairs signed rank test. Correlations were calculated using Spearman’s rank coefficient. P values less than 0.05 were considered significant. Qualitative outcomes of different cohorts of vaccinees were assessed using Chi square test with Yates’ correction. All 95% confidence intervals for proportions were calculated using the Wilson procedure with a correction for continuity (13).

## Results

### Patient and Clinical Characteristics

43 patients with chronic kidney disease (CKD) stage 5 were initially vaccinated with Tozinameran (BNT162b2 BioNTech/Pfizer). Three out of 43 patients developed SARS-CoV-2 infection at 11, 14 and 16 days after the first vaccination, of whom one patient died from COVID-19 infection 19 days after the first vaccination. One patient died from sepsis 37 days after first vaccination (likely unrelated to vaccination and COVID-19) and three patients missed at least one follow-up measurement, thus the remaining 36 patients were included in the study. Of these 34 patients 32 were on hemodialysis and two on peritoneal dialysis. Patients had a median age of 74.0 years (IQR 66.0, 82.0) with a female to male ratio of 11:25. The vaccinated non-dialysis cohort had a median age of 80.0 years (IQR 75.75, 82.25), with a female to male ratio of 30:14. Patient characteristics are summarized in **Table 1**.

**Table 1:** Patient characteristics.

Age	Vaccinated Dialysis Patients (DP, n = 36)	Vaccinated Non-Dialysis Controls (non-DC, n = 44)	Unvaccinated Dialysis Patients (DP, n = 18)	Statistical group difference (p-value)	
				Vaccinated DP *vs* vaccinated-non-DC	Vaccinated DP *vs* unvaccinated DP
Median years (IQR)	74.0 (66.0, 82.0)	80.0 (75.75, 82.25)	63 (45.25, 74.5)	0.226	0.007
Under 50	1	2	6	1.000	0.04
Between 50 and 59	5	2	2	0.234	1.000
Between 60 and 69	10	3	4	0.015	0.751
≥70	20	36	6	0.014	0.148
Male	25	14	12	0.001	1.000
Female	11	30	6	0.001	1.000
**Renal replacement therapy**					
Hemodialysis	34		17	NA	1.000
Peritoneal dialysis	2		1	NA	1.000
Years since start of renal replacement therapy [Median (IQR)]	5 [2, 9]		2 (1, 6.25)	NA	0.034
**Co-Morbidities**					
Hypertension	29	22	15	0.005	1.000
Diabetes mellitus	16	9	7	0.029	0.776
Obesity (BMI >30)	13	5	4	0.14	0.364
Malignancy (recent or history of)	4	8	3	0.532	0.674

NA, not applicable.

### Dynamics of Antibody Levels in Vaccinated Dialysis Patients

**Figure 2:**
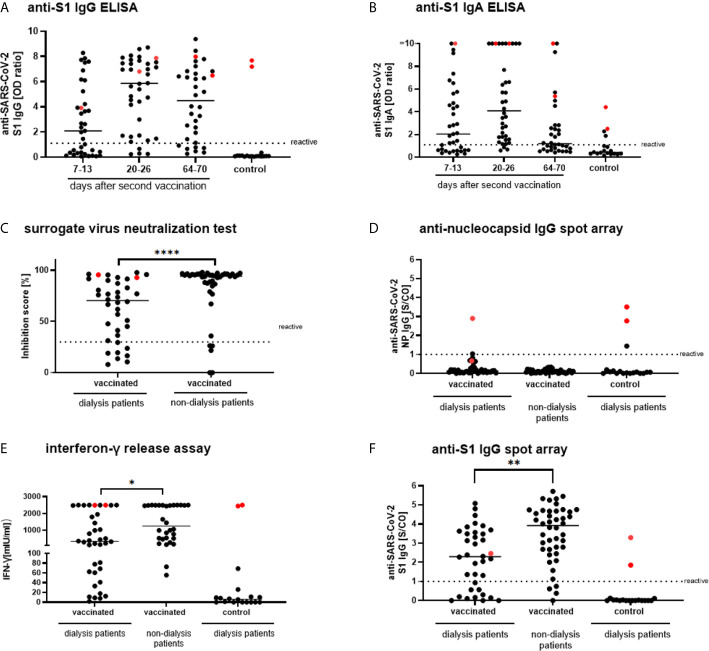
SARS-CoV-2 specific humoral responses in vaccinated and unvaccinated dialysis patients and vaccinated non-dialysis controls. Anti-SARS-CoV-2 S1 IgG and IgA **(A, B)** were measured in serum of dialysis patients on days 7–13 (n = 37), days 20–26 (n = 36) and days 64–70 (n = 32) after a second dose of Tozinameran (BNT162b2) with unvaccinated dialysis patients as controls (n = 18) using a EUROIMMUN ELISA. Participants with a history of PCR-confirmed SARS-CoV-2 infection were shown and marked in red but were excluded from analysis. A surrogate virus neutralization test **(C)** as well as anti-nucleocapsid IgG **(D)** were measured 20–26 days after the second vaccination in dialysis patients, vaccinated individuals of similar age (n = 44), and unvaccinated dialysis patients (n = 18) utilizing the SeraSpot Anti-SARS-CoV-2 IgG assay and Medac cPass. One vaccinated dialysis patient was excluded as the internal assay control failed. **(E)** Whole blood from vaccinated and unvaccinated dialysis patients and non-dialysis individuals was stimulated *ex vivo* with a SARS-CoV-2 S1 peptide pool and IFN-γ concentration in the supernatant was measured by ELISA (borderline 45.92; reactive 91.83). SARS-CoV-2 RT-PCR confirmed patients are shown in red. Anti-S1 IgG **(F)** was analyzed along the same line as the cohorts in **(D, E)**. Horizontal bars depict the median. IgG, Immunoglobulin G; NP, nucleocapsid protein; RBD, receptor binding domain; OD, optical density; S/CO, signal-to-cut-off ratio; IU, international unit. < 0.0001 ****, 0.0001 to 0.001 **, 0.01 to 0.05 *, ≥ 0.05.

One week after the second dose of Tozinameran, 20/36 sera of dialysis patients (55.56%; 95%CI: 38.29–71.67) were reactive for anti-SARS-CoV-2-IgG (**Figure 2A**). Antibody response rate increased to 32/36 patients (88.9%; 95%CI: 73.0–96.4) within three weeks after the second dose and 33/36 sera (91.67%; 95%CI: 76.41–97.82) were also reactive for anti-SARS-CoV-2-IgA (**Figure 2B**). Neutralizing capacity of antibodies was detected in 28/36 (77.78% 95%CI: 60.42–89.28) sera using sNT (**Figure 2C**). Sera were not reactive for anti-SARS-CoV-2-N IgG except for four cases of PCR-confirmed SARS-CoV-2 infection (**Figure 2D**, indicated in red) and one patient of the unvaccinated control group. This patient did not show antibodies against the spike antigens and did not test positive in IGRA. This could be explained by a test artifact or by partial immune response after infection. SARS-CoV-2 specific T-cells, were present three weeks after the second vaccination in 21/31 patients (67.74%; 95%CI: 48.53–82.68) (**Figure 2E**). IGRA correlated positively and significantly (p <0.001) with anti-SARS-CoV-2-S1 IgG (**Supplementary Data, Figure S1**), confirming a specific response of both B and T cells to Tozinameran. For a longitudinal follow-up of vaccinated patients on dialysis we retrieved serum samples from 34 patients ten weeks after the second dose of Tozinameran. The two participants with a history of PCR-confirmed SARS-CoV-2 infection were again excluded from analysis but shown in red throughout the figures. In the analysis, the proportion of dialysis patients reactive for anti-SARS-CoV-2-IgG decreased to 27/32 (84.37; 95%CI: 66.46–94.10) (**Figure 2A**). Along the same line, the frequency of anti-SARS-CoV-2-IgA reactive patients decreased to 19/32 (59.38; 95%CI: 40.79–75.78) (**Figure 2B**).

### Comparison of Antibody Levels to an Age-Similar Non-Dialysis Cohort

Antibody levels of vaccinated patients on dialysis ~3–4 weeks after the second dose were significantly (p <0.01) lower than in the vaccinated control cohort sampled four weeks after the second dose for all four test systems (**Figure 2F** and **Supplementary Data: Figure S2, S3**). In line with this, the percentage of patients with antibody levels above the threshold was higher for the vaccinated control cohort than for patients on dialysis. For fS and RBD antigens these rates differ significantly, with 77.14% (95%CI: 59.44–88.95) and 71.43% (95%CI: 53.48–84.76) for patients on dialysis versus 95.45% (95%CI: 83.29–99.21) and 93.18% (95%CI: 80.29–98.22) for controls, respectively (both p <0.05). For the S1 antigen and the sNT, differences between cohorts were similar, although not statistically significant with 77.14% (95%CI: 59.44–88.95) and 77.78% (95%CI: 60.42–89.28) seropositivity for patients on dialysis versus 90.91% (95%CI: 77.42–97.05) and 87.80% (95%CI: 72.99–95.42) for controls, respectively (p = 0.09 for S1 antigen and p = 0.39 for sNT).

### T-Cell Response to S1 Stimulation in Vaccinated Dialysis Patients

In line with differences in antibody levels, T-cell reactivity to S1 peptide stimulation showed significantly lower levels of interferon-γ release in the dialysis cohort (67.74%; 95%CI: 48.53–82.68) compared to the vaccinated control cohort (93.33%; 95%CI: 76.49–98.84) (p <0.05, **Figure 2E**). Patients not reactive in serologic assays showed negative results for SARS-CoV-2 specific T-cell responses.

## Discussion

We demonstrate a high percentage of SARS-CoV-2 specific seroreactivity after mRNA vaccination with Tozinameran in patients on dialysis. However, the observed response was significantly lower compared to control patients of similar age not on dialysis as well as otherwise reported patients older than 75 years of age (3). Within the longitudinal follow-up time of ten weeks after the second dose of vaccination with Tozinameran, SARS-CoV-2-S1 IgG antibody levels in every 7th dialysis patient remained or decreased to below the threshold for positivity as depicted by pairwise comparison of anti-SARS-CoV-2 IgG responses.

In line with our study on a COVID-19 vaccine, impaired vaccine immunogenicity in patients on chronic dialysis has been reported for those with viruses such as influenza and hepatitis B (14, 15). The decreased number of B and T cells in patients with kidney failure undergoing chronic dialysis might contribute to the differences between patients with KF and without dialysis (16–18). Moreover, impaired clearance of uremic toxins, systemic inflammation, and malnutrition might contribute to lower immunogenicity [reviewed in (19)]. These factors need to be addressed in further studies. However, since patients with KF were excluded from previous COVID-19 vaccine trials, our data present an important report of immunogenicity of COVID-19 vaccines in patients on dialysis.

A recent study from Israel, where 1.2M people have been vaccinated, demonstrated that infection risks vary with patient-specific characteristics (20). These included age and comorbidities, e.g. immunosuppression and type 2 diabetes (20), as risk for a lower immune response. Consistent with that, less robust immune responses were observed in solid organ transplant recipients after one dose of mRNA vaccines (21). Of note, in our dialysis cohort, three patients developed COVID-19 after first vaccination, of whom one died of COVID-19, underlining the need for ongoing non-pharmaceutical intervention after vaccinations, especially in these patients. The occurrence of SARS-CoV-2-S1 IgA in serum after mRNA vaccination of dialysis patients is an interesting observation, as mucosal IgA plays a major role for the protection against respiratory infections (22). If IgA detected in serum after vaccination is part of mucosal immunity is unclear and warrants further studies including longitudinal comparison to non-dialysis cohorts. A rapid decay of SARS-CoV-2 specific IgA antibodies after SARS-CoV-2 has been shown and may be even more pronounced in vaccinated individuals (23).

Limitations of our studies include the small cohort size and the use of only one mRNA vaccine. Therefore, further risk factors contributing to a negative immune response remain to be defined. Gender disparity in our cohort may have contributed to differences in antibody levels although we firmly believe that the advanced age of our participants reduces sex related effects (24). Prospective studies with other vaccines against COVID-19, e.g., viral vector–based vaccines, will help to elucidate the efficacy of different vaccines in these patients. Moreover, antibody titer persistence in patients on dialysis might differ from otherwise healthy persons, which should be addressed in longitudinal observations (25).

Although our data contrast the robust response rate to Tozinameran in the elderly (≥75 years) (3), the overall high percentage of a response three weeks after the second dose, should encourage rapid vaccination of this vulnerable group. Patients on dialysis with and without detectable immune responses need close monitoring and potentially booster vaccinations. Our data demonstrate the urgent need for more detailed studies of the immune response to SARS-CoV-2 vaccines in populations with underlying clinical conditions.

## Data Availability Statement

The original contributions presented in the study are included in the article/**Supplementary Material**. Further inquiries can be directed to the corresponding authors.

## Ethics Statement

The studies involving human participants were reviewed and approved by the Charité ethical review board EA1/068/20, EA4/244/20, and EA4/245/20. The patients/participants provided their written informed consent to participate in this study.

## Author Contributions

ES, LB, KB, FH, VC, and MC planned and designed the study. ES, LB, DH, UW, TS, IL, J-DL, TD, KKa, PT-L, FK, KB, FH, VC, and MC designed the study’s analytic strategy and analyzed the data. MC, AS, KKo, VC, and LS supervised the field activities and data acquisition. ES, VC and MC wrote the first draft of the article. KB, LS, VC, and MC contributed to the critical revision of the manuscript for important intellectual content. All authors contributed to the article and approved the submitted version.

## Funding

Parts of the work were funded by the German Ministry of Research through the projects VARIPath (01KI2021) to VC, and NaFoUniMedCovid19—COVIM, FKZ: 01KX2021 to LS, FK, and VC. VC and ES are participants in the BIH-Charité Clinician Scientist Program funded by the Charité-Universitätsmedizin Berlin and the Berlin Institute of Health. Further parts of the work were funded by a grant from the Sonnenfeldstiftung, Berlin, Germany to AS and KKo and DFG grants (KO 2270/7-1, KO-2270/4-1) to KKo.

## Conflict of Interest

KK is partly contractually provided to Labor Berlin-Charité Vivantes GmbH. VC is named together with Euroimmun GmbH on a patent application filed recently regarding the diagnostic of SARS-CoV-2 by antibody testing.

The remaining authors declare that the research was conducted in the absence of any commercial or financial relationships that could be construed as a potential conflict of interest.
