# Supplementary material for: Immunogenicity of COVID-19 Tozinameran Vaccination in Patients on Chronic Dialysis
Source: Front Immunol. 2021 Jun 30;12:690698. doi: 10.3389/fimmu.2021.690698 (PMC8284337; doi:10.3389/fimmu.2021.690698)
Supplement: Supplementary file 1 [file DataSheet_1.docx]

Supplementary Material

# Supplementary Data

**Supplementary Table S1.** Positive outcome in the different test systems. Percentage with positive result, (95% CI), [no. pos/ no. tested] per cohort*.

| **Cohort** | **Vaccinated**  **dialysis patients** | **Control**  **dialysis patients** | **Control non-dialysis patients** | **Comparison of qualitative outcomes between vaccinated groups** |
| --- | --- | --- | --- | --- |
| **IgG ELISA**  **(1^st^ sampling)** | 55.56 (38.29-71.67)  [20/36] | 0.00 (0.00-24.07)  [0/16] | Not tested | Not tested |
| **IgG ELISA**  **(2^nd^ sampling)** | 88.89 (73.00-96.38)  [32/36] | Not tested | Not tested | Not tested |
| **IgG ELISA**  **(3^rd^ sampling)** | 84.37 (66.46- 94.10)  [27/32] | Not tested | Not tested | Not tested |
| **IgA ELISA S1**  **(1^st^ sampling)** | 61.11 (43.53-76.37)  [22/36] | 12.25 (2.20-39.59)  [2/16] | Not tested | Not tested |
| **IgA ELISA S1**  **(2^nd^ sampling)** | 91.67 (76.41-97.82)  [33/36] | Not tested | Not tested | Not tested |
| **IgA ELISA S1**  **(3^rd^ sampling)** | 59.38 (40.79- 75.78)  [19/32] | Not tested | Not tested | Not tested |
| **NP spot array** | 2.86 (0.15-16.62)  [1/35] | 6.25 (0.33-32.29)  [1/16] | 0.00 (0.00-10.00)  [0/44] | Not tested |
| **RBD spot array** | 77.14 (59.44-88.95)  [27/35] | 0.00 (0.00-24.07)  [0/16] | 95.45 (83.29-99.21)  [42/44] | Yates' chi-square = 4.37  Yates' p-value = 0.04 |
| **S1 spot array** | 74.29 (56.43-86.90)  [26/35] | 0.00 (0.00-24.07)  [0/16] | 90.91 (77.42-97.05)  [40/44] | Yates' chi-square = 2.80  Yates' p-value = 0.09 |
| **Full Spike spot array** | 71.43 (53.48-84.76)  [25/35] | 0.00 (0.00-24.07)  [0/16] | 93.18 (80.29-98.22)  [41/44] | Yates' chi-square = 5.22  Yates' p-value = 0.02 |
| **RBD/ACE2 Inhibition** | 77.78 (60.42-89.28)  [28/36] | 0.00 (0.00-43.91)  [0/7] | 87.80 (72.99-95.42)  [36/41] | Yates' chi-square = 0.75  Yates' p-value = 0.39 |
| **IGRA**  **T-cell reactive** | 67.74 (48.53-82.68)  [21/31] | 0.00 (0.00-24.07)  [0/16] | 93.33 (76.49-98.84)  [28/30] | Yates' chi-square = 4.80  Yates' p-value = 0.03 |

*** Excluding the SARS-CoV-2 RT-PCR- positive patients.

RBD: Receptor-Binding Domain

ACE2: Angiotensin-Converting Enzyme 2

IGRA: Interferon Gamma Release Assay

|  |
| --- |
| **Supplementary Figure S1**  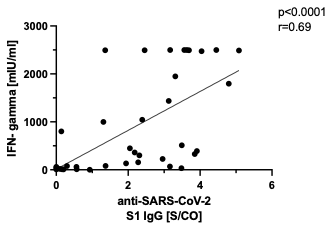  **Figure S1: Correlation of anti-SARS-CoV-2 S1 IgG with IFN-γ concentration after S1 stimulation.** Anti-SARS-CoV-2 S1 IgG were detected in serum of dialysis patients and controls 20-26 days after the second vaccination with Tozinameran by using the SeraSpot anti-SARS-CoV-2 assay and titers independent of the cut-off were correlated with interferon-γ release after S1 stimulation. |
|  |
| **Supplementary Figure S2**  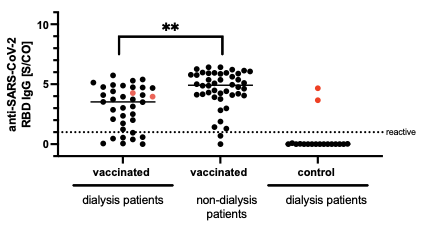 |
|  |
| **Figure S2: Detection of anti-SARS-CoV-2 RBD IgG in vaccinated dialysis patients and non-dialysis patients by SeraSpot anti-SARS-CoV-2 IgG Assay.** Anti-SARS-CoV-2 S1 IgG were detected in serum of dialysis patients 20-26 days after the second vaccination with Tozinameran (n=36) and unvaccinated dialysis patients (n=18) which were compared with vaccinated non-dialysis patients (n=44) by using the SeraSpot anti-SARS-CoV-2 assay. One vaccinated dialysis patient was excluded as the internal assay control failed. SARS-CoV-2 RT-PCR-confirmed patients are shown in red. Horizontal bars depicted the median. S/CO: signal-to-cutoff ratio. < 0.0001 ****, 0.0001 to 0.001 ***, 0.001 to 0.01 **, 0.01 to 0.05 *, ≥ 0.05, not significant, ns. |
|  |
| **Supplementary Figure S3** |
| 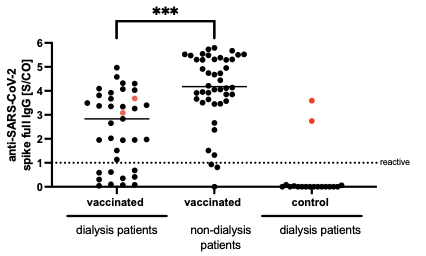 |
| **Figure S3: Detection of anti-SARS-CoV-2 full spike IgG in vaccinated dialysis patients and non-dialysis patients by SeraSpot anti-SARS-CoV-2 IgG Assay.** Anti-SARS-CoV-2 spike full IgG were detected in serum of dialysis patients 20-26 days after the second vaccination with Tozinameran (n=36) and unvaccinated dialysis patients (n=18) which were compared with vaccinated non-dialysis patients (n=44) by using the SeraSpot anti-SARS-CoV-2 assay. One vaccinated dialysis patient was excluded as the internal assay control failed. SARS-CoV-2 RT-PCR-confirmed patients are shown in red. Horizontal bars depicted medians. S/CO: signal-to-cutoff ratio. < 0.0001 ****, 0.0001 to 0.001 ***, 0.001 to 0.01 **, 0.01 to 0.05 *, ≥ 0.05, not significant, ns. |
|  |
| **Supplementary Figure S4** |
| 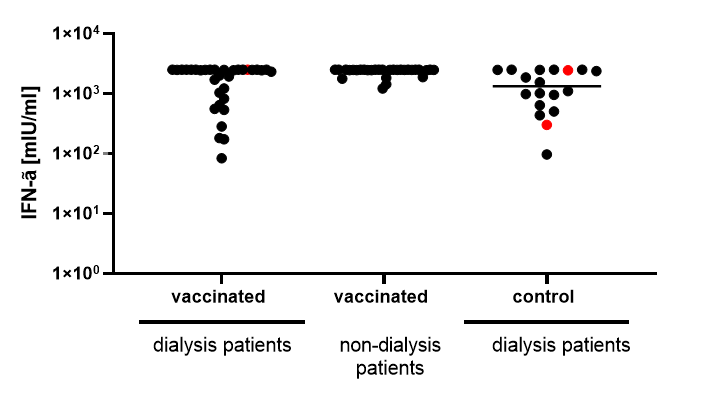  **Figure S4: IFN-γ concentration after mitogen stimulation.** Whole blood from vaccinated dialysis patients (n=33), unvaccinated dialysis patients (n=18) and non-dialysis patients (n=30) was stimulated ex vivo for 24 h with mitogen and IFN-γ concentration in the supernatant was determined by an interferon-γ release assay (IGRA). Horizontal bars depict the median. |

**Supplementary Figure S5**


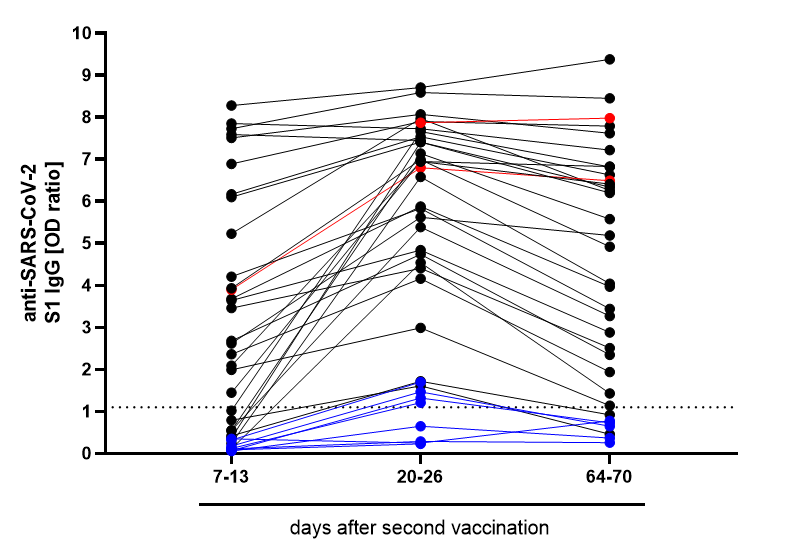


**Figure S5: Pairwise comparison of anti-SARS-CoV-2 IgG responses in vaccinated dialysis patients.** Anti-SARS-CoV-2 S1 IgG were measured in serum of dialysis patients on day 7-13 (n=37), day 20-26 (n=38) and day 64–70 (n=34) after a second dose of Tozinameran using EUROIMMUN ELISA. Patients who were negative in the surrogate neutralization assay are marked in blue. Patients who had a known SARS-CoV-2 infection are marked in red and excluded from analysis.
